# High-performing teams: Is collective intelligence the answer?

**DOI:** 10.1371/journal.pone.0307945

**Published:** 2024-08-12

**Authors:** Luke I. Rowe, John Hattie, John Munro

**Affiliations:** 1 National School of Education, Australian Catholic University, East Melbourne, Victoria, Australia; 2 Science of Learning Research Centre, The University of Melbourne, Parkville, Victoria, Australia; COMSATS University Islamabad, PAKISTAN

## Abstract

**Background/objectives:**

The concept of a general factor of collective intelligence, proposed by Woolley et al. in 2010, has spurred interest in understanding collective intelligence within small groups. This study aims to extend this investigation by examining the validity of a general collective intelligence factor, assessing its underlying factor structure, and evaluating its utility in predicting performance on future group problem-solving tasks and academic outcomes.

**Methods:**

Employing a correlational study design, we engaged 85 university students in a series of complex cognitive tasks designed to measure collective intelligence through individual, group, and predictive phases.

**Results:**

Contrary to the hypothesized single-factor model, our findings favor a two-factor model influenced by Cattell’s theory of crystalized and fluid intelligence. These two factors accounted for substantial variance in group performance outcomes, challenging the prevailing single-factor model. Notably, the predictive validity of these factors on group assignments was statistically significant, with both individual and collective intelligence measures correlating moderately with group assignment scores (*r*_*s*_ = .40 to .47, *p* < .05).

**Conclusions:**

Our research suggests that collective intelligence in small group settings may not be uniformly governed by a single factor but rather by multiple dimensions that reflect established theories of individual intelligence. This nuanced understanding of collective intelligence could have significant implications for enhancing group performance in both educational and organizational contexts. Future research should explore these dimensions and their independent contributions to group dynamics and outcomes.

## Introduction

In 1905 Alfred Binet and Theordore Simon developed the world’s first standardized intelligence test, the Binet-Simon Scale [1, 2]. It was Charles Spearman, however, who significantly advanced our theoretical understanding of intelligence. Spearman introduced the concept of a general intelligence factor, commonly referred to as the *g*-factor. He described this factor in metaphorical terms as a ‘general mental energy,’ denoting the global efficiency of the brain’s cognitive operations. This concept was derived from his empirical observations where he noticed that students who excelled in one academic subject generally performed well across a variety of subjects. From Spearman’s perspective, this ‘general mental energy’ was indeed responsible for the common patterns in performance [3]. Spearman knew that such a phenomenon could, in theory, allow him to predict how students perform on a range of academic tasks based on only a limited sample of their school performance. For example, a student with high grades in their botany class would also be likely to have high grades in different subjects at school (e.g., mathematics, music, languages).

Spearman repeatedly validated his predictions using an early form of correlational analysis (hence the eponymous ‘Spearman’s correlation’). His observations revealed a general trend that the grades of individual students across different subjects were positively correlated–a phenomenon since referred to as a ‘positive manifold’ [4]. From this positive manifold, Spearman was able to mathematically calculate the common variation between the different school grades and extract a *general* factor of intelligence, now referred to by psychologists as the ‘*g*-factor’. Despite its checkered history (e.g., [5]), the role of the *g*-factor in everyday life can hardly be overstated. The influence of general intelligence on academic achievement, job performance, and health outcomes is well-documented and operates through several mechanisms. For example, higher intelligence has been associated with better educational achievement as it enhances learning, problem-solving, and understanding complex concepts, which in turn can lead to higher levels of educational attainment [6]. In the workplace, individuals with higher intelligence tend to learn and adapt faster, which contributes to superior job performance and more rapid development and career progression, as highlighted by Schmidt et al. [7]. Research into the role of intelligence on health, including that by Batty et al. [8], has shown that higher intelligence in early adulthood correlates with lower mortality rates by middle age. They suggest this association may be mediated by better disease management, healthier lifestyle choices, and improved socioeconomic status, all of which are influenced by one’s cognitive abilities.

It could be argued that an equally momentous discovery in the field of group research occurred when, in 2010, Woolley et al. reported that they had observed something analogous to the *g*-factor in human groups [9]. They asked groups of three people to complete a battery of five tests relating to visual puzzles, brainstorming, moral dilemmas, negotiating, and prioritizing. Woolley et al. found positive correlations among all tasks (i.e., a positive manifold) and were able to extract a single factor, referred to as the ‘*c*-factor,’ that was only weakly correlated with the average (*r =* .15) or maximum (*r =* .19) IQ of the group’s individual members. Woolley et al. [9] reported that the *c*-factor accounted for a substantial (43%) proportion of the total variance across the group tasks and exerted a strong predictive effect on a complex criterion task (*r =* 0.52, *p* = .001). Three key variables were found to share a significant relationship with the *c-*factor. First, the variance in speaking turns among group members is thought to predict the *c*-factor in groups, with higher collective intelligence being associated with lower variance (more equal speaking turns) among group members. It is thought that conversational equality promotes a fuller exchange of ideas, enhances collective problem-solving and thus boosts the groups’ *c*-factor. Second, higher levels of the groups’ social perceptiveness, as measured by their average performance on the Reading the Mind in the Eyes (RME) test, is thought to improve communication and cooperation, directly influencing the *c*-factor by facilitating effective group interactions. Third, Woolley et al. claim that females have, on average, higher levels of social perceptiveness, which leads to more effective group dynamics when women are disproportionately represented in the group.

Woolley and colleagues have since conducted follow-up studies attesting to the validity of the *c*-factor in both face-to-face and online settings [10], in laboratory and field settings [11], and across different contexts and cultures [12]. The strongest validation for the *c*-factor comes from a meta-analysis in which Riedl et al. [13] combine results from 22 of their own studies (*N =* 1,356 groups, 5,279 individuals), of which around half have been published. They confirmed a positive manifold across disparate group tasks (finding an average interitem correlation of 0.27 [0.12 to 0.50]), an excellent fit using a meta-analytic confirmatory structural equation modelling, and an average variance explained by the *c*-factor of 44% across the battery of group tasks (p. 2). A major limitation of this meta-analysis was that it was not exhaustive and only included studies from the MIT online collective intelligence battery; it excluded other published studies of tests of collective intelligence–most of which depart from the findings and conclusions proffered by Woolley and colleagues.

Other reviews have found mixed evidence of a *c-*factor in groups. For example, Graf-Drasch et al. [14] found different group-IQ tests led to systematic differences in the factor structure of collective intelligence and argued that the collective intelligence factor applied only to *well-structured tasks* such as logical puzzles and numerical reasoning. In contrast, *ill-structured tasks* were found to have a multidimensional factor structure that depended on whether groups engaged in activities requiring decision-making, conflict, mixed motives, planning, and creativity.

Rowe et al. [15] performed two meta-analyses comparing the predictive effect of the *c-*factor with the average IQ scores of individual members in groups. The first meta-analysis found a weak to moderate correlation (*r =* .26) between the *c-*factor and group criterion tasks. The second meta-analysis found little to no relationship (*r* = .06) between individual IQ scores and group criterion tasks, but only included five independent samples (*k* = 5), so was unlikely to produce a reliable estimate of the effect. Consequently, despite results seemingly favoring collective over individual intelligence in accounting for variation in group performance, Rowe et al. [15] were careful to emphasize that such conclusions are premature in light of methodological shortcomings and a general paucity of independent research on the topic.

If collective intelligence does indeed emerge in small groups engaged in complex problem-solving tasks and is shown to exert a substantial influence on group problem-solving and performance, then it is likely to be of enormous theoretical and practical import. The implications are obvious: One cannot otpimize group performance unless one knows what factor/s play a significant role in these situations. If group problem-solving, learning, and performance operate as a function of the groups’ collective intelligence, irrespective of the intelligence of the groups’ individual members, then researchers and practicioners should pay close attention to the factors that optimize the collective intelligence of the group. Assessment and evaluation of group performance, including project and work teams in occupational setttings, group presentations and written assignments in educational settings, for example, would need to be concieved in light of the underlying collective intelligence of the groups, building teams and allocating members accordingly. Theories of group learning, collaborative problem-solving, and team effectiveness that overlook collective intelligence would be grossly incomplete–potentially failing to acknowledge what could be *the* dominant factor driving outcomes in these domains. In sum, the *c*-factor could do for group research what the *g*-factor has done to transform research on indiviudal differences.

### Aim and research questions

We aim to explore the role of collective intelligence among small groups engaged in a variety of intellectually challenging tasks. We draw upon the methodology outlined in Woolley et al. [9]. Specifically, our first research question seeks to explore the validity of Woolley et al.’s claim that a single collective intelligence factor emerges in small groups in a way that is distinguishable from the intelligence of their individual members. We explore the factor structure of this phenomenon using a range of theoretically plausible structural equation models. The first model we seek to test will be the *c-*factor proposed in the original study by Woolley et al. [9]. If this model provides the best fit, it will be interpreted as having replicated the *c-*factor. The second model we evaluate is based on Bates and Gupta’s [16] studies where average individual IQ scores “accounted for around 80% of group-IQ differences” (p. 46). If this model provides the best fit, it will undermine the *c-*factor by suggesting that it is a special case of individual intelligence ‘writ large.’ The third and final model we test with SEM will be guided by the results from exploratory factor analysis and the use of Horn’s parallel analysis which provides an interpretative benchmark where eigenvalues above a simulated threshold are included on the basis that they are larger than those expected by chance [17]. This latter model is not constrained to a single factor, so it has the advantage of being free to produce a multidimensional factor structure. In such a case, superior fit could indicate a domain-specific rather than domain-general factor structure behind group performance, challenging the single-factor models proposed by Woolley et al. [9] and Bates and Gupta [16].

- *Research Question 1*. Does the single general factor of collective intelligence proposed by Woolley et al. [9] provide a better explanation for the observed data compared to alternative explanations?

Our second question relates to the antecedents of collective intelligence. The original study by Woolley et al. [9] proposed three main variables are causally responsible for collective intelligence in groups. The first of these was the distribution in speaking turns among group members, measured using the SD of speaking turns, and showed that groups performed worse when fewer members shared in conversational exchanges. The second causal influence was the proportion of females in the group: As the proportion of females in the group increased, so too did *c-*factor scores. Thirdly and relatedly, greater social perceptiveness, as demonstrated on the Reading the Mind in the Eyes (RME) test [18], which measures the ability to accurately infer mental states using only the eyes-section of a human face, were positively associated with the collective intelligence of the groups and mediated the positive role played by the proportion of females in the group (females, on average, have better RME scores compared to males).

- *Research Question 2*. What role do the three key variables identified by Woolley et al. [9] play in facilitating collective intelligence, including conversational turn-taking, the proportion of females, and social perceptiveness?

The next question relates to the predictive validity of the *c-*factor on complex group tasks. Specifically, we explore whether the *c-*factor can predict group performance on the Moon Landing Exercise (MLE). It involves groups imagining themselves to be a space crew stranded on the moon. Participants must justify the use of 15 items (e.g., box of matches, stellar map) that are to be used to aid survival on a trip back to their mothership located 200 kilometres away. The MLE has been used in previous group performance research [19] and was selected because it provides an analogue to the Desert Survival Scenario (DSS) used by the original research team to test the predictive validity of the *c-*factor where it was “a strong positive predictor of performance on the task” [12]. The MLE and DSS are methodologically identical but provide different survival scenarios.

- *Research Question 3*. Does the *c-*factor predict the groups’ performance on the complex criterion task?

Our final question relates to the external validity of the *c-*factor in the context of the student-participants’ group assignment scores. Given that the *c-*factor is expected to have a positive effect on group performance, it is reasonable to expect that small groups that demonstrate higher levels of collective intelligence would also maintain attributes that generalize to other group tasks (this is indeed the claim made by Bates and Gupta [16]). We seek to better understand this by testing the relationship between the *c-*factor and group assignment scores provided to the researchers by the participants. This led us to ask the following:

- *Research Question 4*. Does the *c-*factor predict grades received by participants for university-level group assignments?

## Methods and materials

### Participants

Participants were recruited from an Australian university over 6-weeks using physical and electronic flyers and university notice boards across two faculties that were linked to the lead author’s bi-faculty enrolment for a doctoral program (education and psychology). The final sample involved 85 university students (*M*age = 25.21 years, 96.47% studying full-time, 71.76% female, and 83.53% born overseas) who were allocated to one of 29 groups (*Mgroup-*size = 2.93 people, group-size range: 2–5 people). A more detailed breakdown of participant demography can be seen in the supplementary files (S3 File). It should be noted that these demographic characteristics were typical of the education and psychology faculties at the university, where a higher proportion of female and overseas born students are enrolled in postgraduate programs. Written informed consent was obtained from participants. Students had to be actively enrolled at the university to be part of our study, as we corresponded via student email and notice boards. English-speaking and reading fluency was required for inclusion in the study, and these skills were screened during a series of practice tests prior to the experimental tasks. It was explained to participants in writing and spoken word that they were free to withdraw from the study at any time. No prospective participants withdrew or failed the screening tasks, and so no data was missing from the final dataset. All participants were compensated with a $20 AUD voucher. An opportunity to debrief was provided upon completion.

This study was approved by and conducted in compliance with the university’s Human Research Ethics review board as a part of the *Problem-solving in Small Groups* project (March–November 2016), and personally signed-off by the Chair of the Melbourne Educational Research Institute (MERI) Ethics Advisory Group.

### Procedures

**Fig 1:**
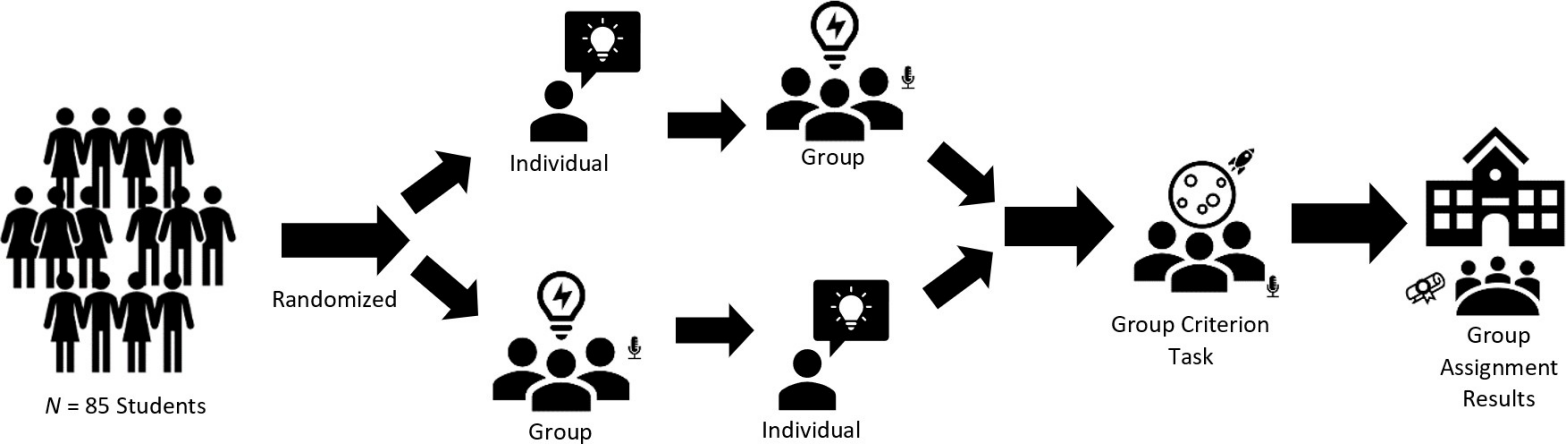
Flow chart of the study. This shows the three major phases of the study (individual, group, and prediction).

We used a within-subjects, correlational design with three major phases: an individual phase, a group phase, and a prediction phase (Fig 1). The materials and methods used across each of these phases were informed by paradigm adopted by Woolley and colleagues, including our primary and secondary measures, as well as our outcome variables. Power calculations for the sample size estimate were benchmarked against the correlation of *r =* .52 between the *c-*factor and the criterion task reported by Woolley et al. [9]. We contextualized this effect using Cohen’s [20] conventions of statistical power set at 80% (1 –*β*) with an α of .05 with the ‘pwr’ package version 1.3–0 in R [21]. We calculated a minimum sample size of 26 groups using a two-sided test or 21 groups using a one-sided test. According to these assumptions, our observed power to detect an effect, if were present in the population, was ~ 85%.

The individual phase involved participants answering demographic questions before completing individual IQ and Reading the Mind in the Eyes tests, along with various measures for our secondary analysis (Table 1).

**Table 1:** Instruments and measures for individual participants (Individual phase).

Instrument	Domain	Description	Source / Reference
*Primary Measures*			
Demographic variables (Sex)	Sex differences	Questionnaire with self-report items completed individually using pen and paper (no time limit)	N/A
The 16-Item International Cognitive Ability Resource (ICAR) Sample Test	Intelligence (Verbal Reasoning, Matrix Reasoning, Letter-number reasoning, and Three-Dimensional Rotation)	16 multiple choice test items (0 Incorrect or 1 Correct) administered to individuals in a group (classroom-style) setting. Completed using pen and paper. This test has reported correlations of .81 and .82 with composites A and B of the Shipley-2 (see Condon & Revelle, 2014); a brief measure of crystallized and fluid cognitive ability that itself has reported correlations with the Wonderlic Personnel Test and the Wechsler Adult Intelligence Scale or WAIS-III upwards of .72 and .85 respectively (Shipley et al., 2009). The ICAR-Sample Test was used to operationalize the construct of individual IQ and includes a sample of four questions pertaining to: Letter and number reasoning, Matrix reasoning, Verbal reasoning, and Three-dimensional rotation (Condon & Revelle, 2014). (15 minutes)	Condon and Reveille [22].
The Eyes Test—36 Item Adult Version	Social perceptiveness (recognizing emotions with limited visual cues)	36 multiple choice test items (0 Incorrect or 1 Correct) administered to individuals in a group (classroom-style) setting. Completed using pen and paper (10 minutes)	Baron-Cohen et. al., [18].
*Secondary Measures*			
Additional Demographic Information	Age, nationality, main language, educational attainment, occupation, ethnicity	Questionnaire with self-report items completed individually using pen and paper (no time limit)	N/A
Brief Emotional Intelligence Scale 10 or BEIS-10	Emotional Intelligence Trait Markers	Questionnaire with self-report items completed individually using pen and paper (no time limit); used Likert-type scale, 1 = Strongly Disagree, 5 = Strongly Agree	Davies et al. [23].
The 50-item IPIP version of the Big Five Markers	Personality (Big 5)	Questionnaire with self-report items completed individually using pen and paper (no time limit); used Likert-type scale, 1 = Very Inaccurate, 5 = Very Accurate	Goldberg [24, 25].

*Note*. All instruments were administered to individuals using separate A4 paper booklets in the order they appear above (upper to lower rows).

The group phase, which was randomly counterbalanced with the individual phase, consisted of a group IQ test battery involving 18 closed- and 5 open-ended vocabulary questions, a group ideation task, 15 matrix reasoning items, and 10 collective attention and memory questions based on a short film. Multiple-choice items were evaluated using automated scoring, while written answers were independently rated by two researchers using a standardized rubric. Answers from the two open-form subtests, group brainstorming and the open-form vocabulary measure, generated an overall agreement of 97%, with only a few disagreements flagged then resolved through discussion. As with Woolley et al. [9], we also included group process and emergent variables (Table 2).

**Table 2:** Instruments and measures for groups (Group phase).

Subtest	Domain	Description	Source / Reference
*Group IQ Subtests*			
Subtest 1: Group Vocabulary (Multiple Choice)	Group Verbal Reasoning & Vocabulary Recognition	18 multiple-choice vocabulary items sampled from the Educational Testing Service’s ‘Kit of Factor-Referenced Cognitive Tests’, Advanced Vocabulary Test II–V-5, part 2 (4 minutes)	Ekstrom, et al. [26]
Subtest 2: Group Vocabulary (Open Form)	Group Verbal Reasoning & Vocabulary Recall	5 open-form advanced vocabulary words to be defined from a selection of the Mill Hill Vocabulary Scale (MHVS) (5 minutes)	Styles, et al. [27] and Raven [27].
Subtest 3: Group Matrices	Group Matrix Spatial Reasoning & Pattern Recognition	15 items selected from Raven’s Standard Progressive Matrices–Plus (SPM+) Version (final five items from sets C, D, and E) (10 minutes)	Styles, et al. [27] and Raven [27].
Subtest 4: Group Brainstorming	Group Creativity, Ideation, and Divergent Thinking	1 group-brainstorming stimulus problem requiring as many relevant, novel ideas as possible in 5 minutes (e.g., ‘What benefits and difficulties would arise if everyone born after 2003 had an extra thumb on each hand?’)	Brophy [28] and Paulus and Dzindolet [29].
Subtest 5: Group Memory & Attention	Group Memory and Attention (transactive memory—recognition)	10 multiple-choice questions aimed at measuring the group’s memory and attention, and developed for the present study relating to a five-minute section of a film known as ‘Big Buck Bunny’ (10 minutes, including 5 mins on stimulus and 5 mins on multiple choice questions)	Methods by Woolley et al. [9] and video by Goedegebure et al. [30].
*Emergent (derived) and Group-Process Measures*			
Friendship	Group with nil, partial, or full member friendship (prior to participation)	Researcher recorded friendship / affiliation status on the enrolment records prior to completing the experimental procedures (0 = Non-Friend, 1 = Part Friendship, 2 = Full Friendship).	Chung et al. [31].
Communication	Group Conversational Turn-taking and Word Count	All communication was recorded via lapel microphones per participant, converted to a WAV file, and subsequently transcribed by a professional transcription service to a written document where individual turns and total words were counted and aggregated to group-level	Woolley et al. [9].
The Situational Motivation Scale (SIMS).	Motivation (Situational and Intrinsic Motivation, Amotivation and Extrinsic Motivation)	Questionnaire with self-report items completed individually using pen and paper (no time limit); used Likert-type scale, 1 = Corresponds Not at All, 7 = Corresponds Exactly	Guay et al. [32].
The Six Item Perceived Cohesion Scale (PCS) for small groups	Group Cohesion (Perceived Group Cohesion with Group Members)	Questionnaire with self-report items completed individually using pen and paper (no time limit); used Likert-type scale, 1 = Strongly Disagree, 7 = Strongly Agree	Chin et al. [33].
The Group Task Satisfaction Scale	Group Task Satisfaction (abstracted at the group level)	Questionnaire with self-report items completed individually using pen and paper (no time limit); used Likert-type scale, 1 = Strongly Disagree, 7 = Strongly Agree	Mason and Griffin [34, 35].

*Note*. All group IQ subtests were administered to groups via an A3 poster-style booklet containing all items in the order they are presented (upper to lower rows).

Finally, the predictive validity of the *c*-factor is evaluated based on the correlation between the group-IQ scores and the Moon Landing Exercise. The Moon Landing Exercise was identified as an appropriate task for external validation because it draws upon multiple domains of group performance spanning several aspects of McGrath’s [36] task circumplex (e.g., planning, decision-making, creativity, conflict, mixed motives), thereby integrating multiple performance domains otherwise isolated in each of the separate group-IQ subtests. Moreover, results on this type of task have been strongly linked to collective intelligence [12].

External validity was evaluated based on the correlations between the individual and group-IQ scores, and the group assignment scores provided by the participants in our study. A total of 30 participants representing 36.6% of our original sample volunteered to share their most recent group assignment result/s with us via screenshots and/or documented evidence such as photographic evidence of their academic transcript. It should be noted that the researchers did not otherwise inspect group assignment scores on the students’ academic transcripts as the ethics were approved only if results were voluntarily provided by the students. Participants who declined participation did so mostly on the basis that they had no group assignment results to report for that semester (34 students or 41.5%), did not feel comfortable sharing their personal results (5 students, 6.1%), or did not respond to the email request (13 students or 15.9%). Of the 30 students who provided group assignment scores, 9 were able to provide 2 or 3 scores; in this case we used the statistical mean to represent their overall group assignment result (Table 3).

**Table 3:** Instruments and measures for criterion tasks (Predictive phase).

Subtest	Domain	Description	Source / Reference
*Group IQ Subtests*			
Moon Landing Exercise (MLE) (predictive validation)	Multiple domains (e.g., negotiation, prioritization, judgement, and decision making)	The MLE involves a hypothetical situation in participants imagine themselves to be a space crew stranded on the moon 200 kilometres from the mothership. Among the salvaged wreckage of their explorer craft are 15 items (e.g., box of matches, stellar map, signal flares) that are to be ranked in order of declining contribution to survival on the trip back to the mothership. Because this was a judgemental rather than intellective task (see Laughlin, 2011), it involved soliciting answers from groups that were probabilistic in nature rather than correct or incorrect per se. Overall scores were based on how well groups compared to expert rankings; higher marks were given to groups that more closely approximated expert rankings and lower marks when rankings differed from experts (6 minutes)	Items were adapted to an Australian testing context from Knox [37].
Group Assignment Score (external validation)		Scores provided by the students voluntarily via follow-up email several weeks after their participation in the experiment. Those who consent to this phase of the study will be asked to provide a screenshot or an objectively verifiable document displaying their most recent group assignment grade/s	N/A

*Note*. All group IQ subtests were administered to groups via an A3 poster-style booklet containing all items in the order they are presented (upper to lower rows).

## Results

### Research question 1. How does Woolley’s c-factor compare to alternative models of group performance?

**Fig 2:**
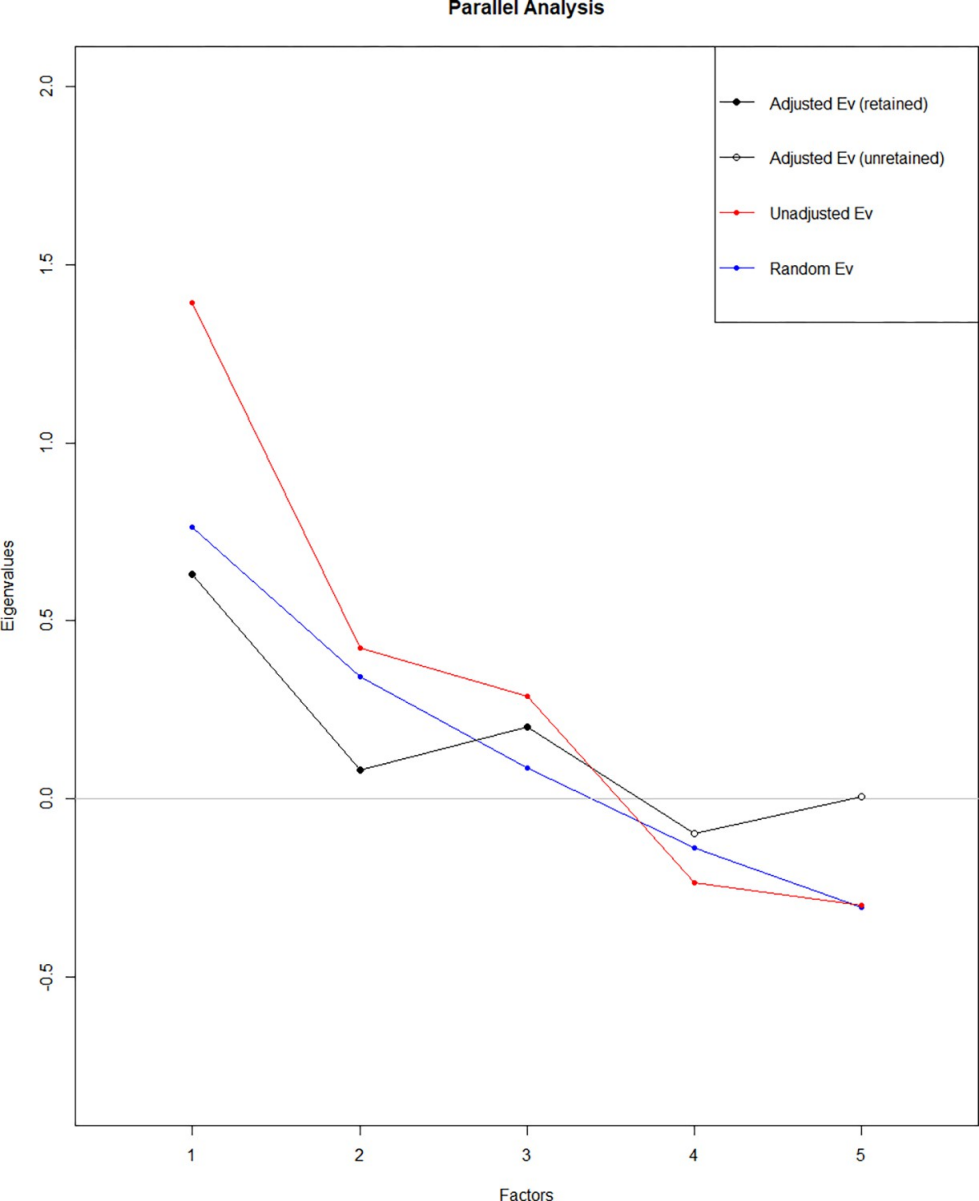
Horn’s parallel analysis and scree plot. *Note*. The three adjusted eigenvalues > 0 are retained (black line, filled dots) and < 0 are unretained (black line, empty dots). For comparison, the average random eigenvalue is indicated by the blue line, while the unadjusted eigenvalues from the observed data are indicated by the red line.

The first research question (RQ1) compared the *c-*factor with alternative explanations of variation in group performance based on model fit and theoretical plausibility. We used exploratory factor analysis with principal axis factoring and oblique rotation (Promax) in the ‘paran’ package version 1.5.2 in R ([38], see Dinno, [Unpublished]). This allowed us to generate a Scree plot and conduct Horn’s parallel analysis as an objective benchmark to evaluate which eigenvalues were larger than those expected by chance [17]–methods in keeping with Woolley et al. [9]. Results revealed that the scree plot had two ‘elbows’ that did not level-off until after the third factor, and the parallel analysis output suggested 3 factors should be retained (Fig 2). These accounted for 41%, 22%, and 20% of common variance respectively. Therefore, our initial results suggested a single *c-*factor model was not the most plausible fit for the data.

**Fig 3:**
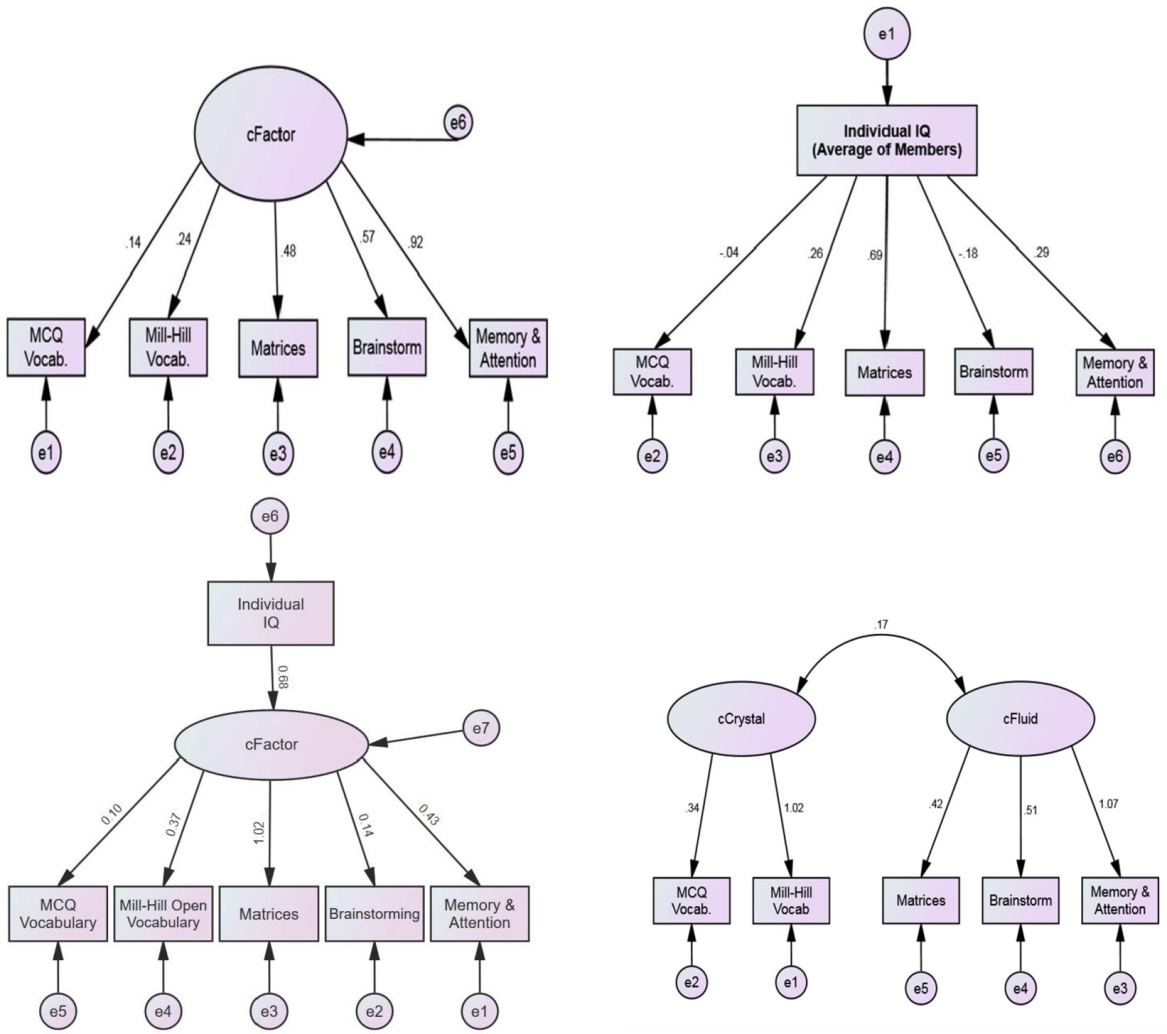
Comparing structural equation models. **A:** Single cFactor SEM resembling Woolley et al., (*χ*^*2*^ *(5) = 10*.*251*, *p =* .*068*, *GFI =* .*883*, *CFI =* .*694*, *RMSEA =* .*194)*. **B:** Individual IQ indirectly accounts for Group-IQ via latent cFactor resembling Bates and Gupta model *(χ*^*2*^ *(9) = 21*.*649*, *p =* .*010*, *GFI =* .*824*, *CFI =* .*652*, *RMSEA =* .*224)*. **C:** Individual IQ directly accounts for Group-IQ *(χ*^*2*^ *(10) = 27*.*731*, *p =* .*002*, *GFI =* .*746*, *CFI =* .*512*, *RMSEA =* .*252)*. **D:** ^*a*^ *c*Fluid-*c*Crystal SEM resembling Cattell’s Gf-Gc theory *(χ*^*2*^ *(4) = 6*.*926*, *p =* .*140*, *GFI =* .*914*, *CFI =* .*829*, *RMSEA =* .*162)*. *Note*. Figures compare SEMs explaining variance across all five Group-IQ subtests. All models were generated using SPSS AMOS graphics v27. ^*a*^Model selected for subsequent analysis.

The next step used exploratory structural equation modelling [39] to test the fit of the *c-*factor in relation to the observed and estimated covariance matrices. Fit statistics generated by these analyses can be misleading even when indices represent ‘excellent’ fit. This is because any number of alternative models can demonstrate equally good or better fit or show no significant degradation to fit indices for a more parsimonious model [40]. For this reason, it is advisable that model fit be compared across several theoretically plausible alternative models [41]. The alternative models can be seen in Fig 3.

The first of these models draws upon the theoretical structure proposed by Woolley et al. [9] in which a single, general factor (i.e., the *c-*factor) of collective intelligence has a positive, direct, and differential effect on the groups’ performance across the full range of five subtests (Fig 3A). The second model (Fig 3B) is based on Bates and Gupta’s [16] solution which suggests that individual IQ accounts for group-IQ via the latent *c-*factor. In Bates and Gupta’s [16] model, average individual IQ scores “accounted for around 80% of group-IQ differences” (p. 46). Though we continue using the SEM abbreviation for the third model (i.e., Fig 3C), path diagrams are used to depict correlations between the average individual IQ scores in each group and the raw scores for each of the five group-IQ subtests. Unlike the genuine SEMs, this method reveals total collinearity between test items and not just the common variance used in a latent model.

Finally, for Fig 3D we examine what has been described in psychometric theory as the crystal-fluid model of intelligence. Horn and Cattell [42] originally distinguished fluid from crystallized intelligence by characterizing the former as a “measurable outcome of the influence of biological factors” and the latter as the “principal manifestation of a unitariness in the influence of experiential-educative-acculturation influences” (p. 254). Psychometrically, tests of fluid intelligence typically assess working memory, processing speed, and inductive and deductive reasoning, while tests of crystal intelligence typically assess general knowledge, vocabulary, and culturally relevant facts [43].

According to modern standards for statistical fit [44], the model which best fit the data was the *c*Fluid-*c*Crystal model (Fig 3D) while the other three models, including the *c-*factor model proposed by Woolley et al. [9] and the individual IQ model proposed by Bates and Gupta [16], showed inferior fit. We retained Fig 3D, the *c*Fluid-*c*Crystal model, for all subsequent analyses because it showed the best statistical fit and was consistent with prior theory evinced in the fluid-crystal (Gf-Gc) model of intelligence proposed by Cattell [45, 46]. We also note that both *c*Fluid and *c*Crystal factors shared strong (*r* ≥ .54) correlations with the group-IQ composite but were themselves not strongly correlated (*r* = .25), and mirrored the average correlation between all five group-IQ subtests (*r* = .26, representing a ‘positive manifold’). These trends may suggest the possibility of independent contributions made by each of the *c*Fluid and *c*Crystal factors to the overall collective intelligence of the group. Therefore, our results were inconsistent with Woolley et al.’s [5] notion of a single general collective intelligence factor in groups. We now turn to answering research questions 2 to 4 in light of the *c*Fluid-*c*Crystal model presented in Fig 3D.

### Research question 2. What are the correlates of collective intelligence?

Variance in speaking turns, the group’s average levels of social perceptiveness, and the group’s proportion of females were among the strongest predictors of collective intelligence in the original and subsequent studies by Woolley and colleagues. Moreover, the average IQ of the individual members of each group did not contribute toward the groups’ performance on the group-IQ tests in any substantive way. Our analysis includes the group-IQ composite which is factor-neutral, and the *c*Fluid-*c*Crystal model of collective intelligence. The group-IQ composite score was comprised of the total raw-score that each group achieved across a battery of group-IQ subtests involving 49 items. Groups could achieve scores well beyond 49 points, however, because one of the items (Group brainstorming) was an open-ended task and points were awarded for each unique response in a 5-minute span of time. We chose to retain the group-IQ composite throughout our analyses to offer a factor-neutral option against which predictor variables could be assessed. This overcomes some of the limitations of previous research where the hypothesized factor structure is assumed to be a valid representation of the latent intelligence of the group; yet reviews of the literature and the present findings suggest this issue remains an ongoing debate. Including the raw group-IQ scores affords readers the opportunity to by-pass this debate and judge the validity of the relationships between predictor variables and criterion tasks on the merits of their observable–rather than contested latent–characteristics. Given that the *c*Fluid-factor was the first of those retained in our model and explained the most variance, it can be considered as the factor that most closely resembles the single *c*-factor proposed by Woolley and colleagues (model constraints notwithstanding). Thus, our analysis explored whether the main predictors of collective intelligence hypothesized by Woolley et al. [9] were significant predictors of the raw group-IQ composite and the model of collective intelligence proposed in the present study (*c*Fuid-*c*Crystal). Bivariate (Pearson’s) correlations for all variables in our primary and secondary analyses are displayed in Table 4.

**Table 4:** Correlations and descriptive statistics.

		1	2	3	4	5	6	7	8	9	10	11	12	13	14	15	16	17
	*Outcome Variables*																	
1	*Group IQ*	1.		* *														
2.	*cFluid*	.671^**^	1															
3.	*cCrystal*	.540^**^	.*252*	1														
*Primary Predictor Variables (per Woolley et al*., *2010)*																		
4.	Individual IQ^a^	.241	.294	.266	1													
5.	Social Perceptiveness^a^	.265	.092	.129	.345	1												
6.	Speaking Turns (SD)	.186	.060	.007	-.219	-.236	1											
7.	% Female	-.168	.054	-.097	-.165	-.180	-.068	1										
*Secondary Predictor Variables*																		
8.	Max IQ	.295	.323	.217	.770**	.105	.163	-.254	1									
9.	Extraversion^a^	.241	.018	.205	.202	.167	.186	-.278	.182	1								
10.	Agreeableness^a^	.215	.174	.212	.190	.378	-.053	.008	.023	.403	1							
11.	Conscientiousness^a^	.389*	.308	.483**	.143	-.004	.097	-.288	.185	.442	.540**	1						
12.	Neuroticism^a^	.048	.073	.187	.334	.206	-.195	-.272	.333	.234	.573**	.316	1					
13.	Openness^a^	.337	-.054	.265	.158	.311	-.062	-.302	.109	.686**	.159	.367	.072	1				
14.	EQ (trait)^a^	-.077	-.253	-.099	-.070	.130	-.196	.147	-.182	.051	.213	-.022	.222	.118	1			
15.	Motivation^a^	-.263	-.188	-.250	-.269	-.615**	.196	.131	-.126	-.002	-.079	.133	-.147	-.153	.067	1		
16.	Cohesion^a^	-.083	-.118	-.015	.018	.357	-.572**	.013	-.274	.219	.416*	.154	.272	.218	.407*	-.200	1	
17	Satisfaction^a^	.053	.028	-.133	-.046	.436	-.449	-.071	-.314	.088	.440*	.093	.250	.061	.466*	-.175	.804*	1
	Mean	39.86	0.00	0.00	9.10	25.57	28.26	70.46	11.24	30.64	39.35	34.98	30.29	34.89	37.83	64.13	35.42	67.07
	SD	6.80	1.00	1.00	2.01	2.90	25.81	29.93	2.12	5.27	3.67	4.89	5.39	5.17	3.56	6.89	3.75	6.68

*Note*. Italicized variables (1, 2, and 3) are outcome variables; cCrystal = The factor weights of crystallized intelligence subtests (Ekstrom’s advanced multiple-choice vocabulary, Mill-Hill Open-ended Vocabulary Scale); cFluid = The factor weights of fluid intelligence subtests (Group Brainstorming, Raven’s Matrices, Group Memory & Attention) SD = Standard Deviation. Social perceptiveness = Social perceptiveness as measured by average performance on the ‘Reading the Mind in the Eyes’ test; Speaking Turns (SD) is the standard deviation score for the number of conversational speaking turns taken by group members during the group-IQ tests; EQ = Trait-based emotional intelligence; Group IQ = Raw composite scores from the battery of five subtests used in the collective intelligence test battery described in Table 2; % Female = The proportion of the total number of group members that self-identify as being female; Max IQ = The group’s highest-IQ member; Extraversion, Agreeableness, Neuroticism, Conscientiousness, and Openness represent domains of the ‘big 5’ personality profile; Motivation = Situational Intrinsic-Extrinsic Motivation Scale; Cohesion = Perceived Cohesion Scale; Satisfaction = Group Task Satisfaction Scale.

^a^ Correlations based on group averages (statistical mean per group).

*P ≤ .05

**P ≤ .01

Neither the correlation of variance in speaking turns (*r* = .19, *p* = .33), the groups’ average social perceptiveness scores (*r* = .27, *p* = .16), nor the proportion of females in the groups (*r* = -.17, *p* = .38) showed any significant correlation with the group-IQ composite scores. When these same predictor variables were correlated with the *c*Fluid factor, no values exceeded *r* ≥ .10 and none were statistically significant (*p* < .05). When correlated with the *c*Crystal factor, no values exceeded *r* ≥ .10 except for the group’s average social perceptiveness (*r =* .13) and none were statistically significant (*p* < .05).

**Fig 4:**
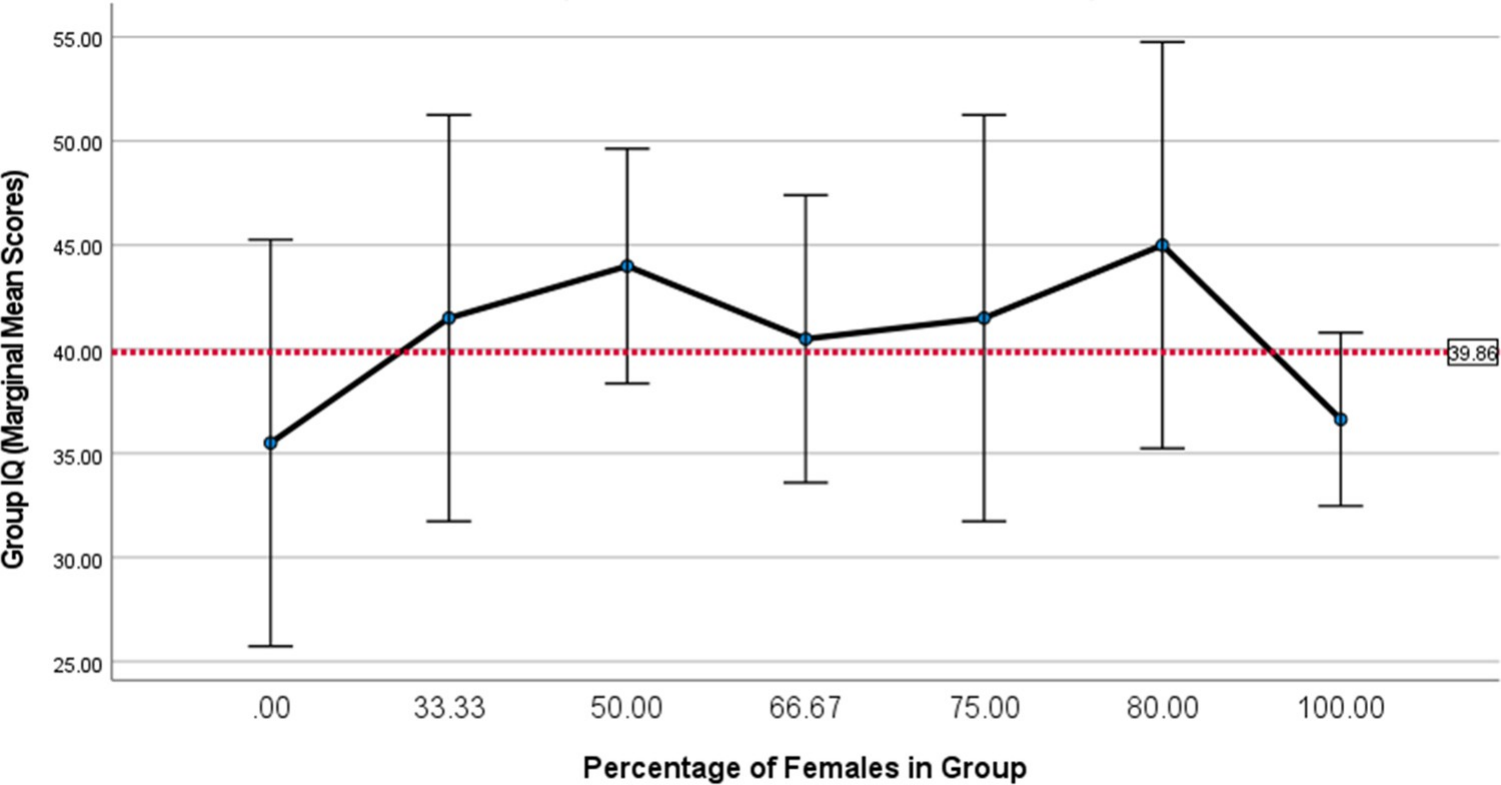
Percentage of females and group-IQ. *Note*. One-way ANOVA results display mean group-IQ scores (error bars 95% CI) across seven categories ranging from the lowest (.00) to the highest (100.00%) proportion of females. Proportions were a direct function of group size: either 2, 3, 4, or 5 participants. The dotted-line represents the grand statistical mean for group-IQ for all groups (*N = 85 participants*, 29 groups).

A well-publicised finding in Woolley et al. [9] stems from the claim that a higher proportion of women improves the groups’ collective intelligence [47, 48]. We therefore performed a robustness check using one-way ANOVA, which corroborated our correlational analysis by revealing that the proportion of females in the groups had no discernible effect on the group-IQ composite performance, *F*(6, 22) = 1.2, *p* = .34 (Fig 4).

Taken together, these results suggest that there is little to no relationship between collective intelligence and the three most important candidate causes proposed by Woolley et al. [9], namely, conversational turn-taking, the proportion of females in the group, and the group’s average social perceptiveness as measured by the Reading the Mind in the Eyes test.

Because contradictory claims exist about the role of the *c* and *g* factors in group performance (e.g., [5] vs. [12]), we conducted further analyses to account for the role of individual intelligence. We did this by extracting a *g*-factor from our individual cognitive ability measure [22] and explored its relationship with each of the five subtests in the group-IQ test battery. Group members with the highest IQ, the average IQ of individual group members, and the groups’ average *g* loadings (derived from averaging the *g* loadings of individual group members) each had significant (*p* ≤ .001) and strong correlations with the group matrices, *r =* .69, 69, and .60, respectively. This suggests that the effect of intelligence measured at the individual-level is more apparent at the group-level when the tests used are the same or at least validated around the same theoretical construct (e.g., psychometric *g*). It may also indicate that the alternative *c*Fluid-*c*Crystal model we propose is itself a group-level construct that reflects the structural features of the intelligence of the group’s individual members (Gf-Gc writ large).

### Research question 3. Predicting performance outside of the group-IQ test

The third research question (RQ3) explored the predictive validity of collective intelligence when operationalized using either the group-IQ composite, the *c*Fluid, or the *c*Crystal loading scores. In our study, the criterion task used was the Moon Landing Exercise, which was administered with a brief (5-minute) delay following the group-IQ test battery. Correlations were weak and not statistically significant between the Moon Landing Exercise and either: group-IQ composite, *r* = .11, *p* = .56; *c*Fluid, *r* = .10, *p =* .59, or; *c*Crystal, *r* = .04, *p =* .84. A series of simple linear regressions were conducted to examine the relationships between Woolley’s *c*Factor, *c*Fluid, *c*Crystal, and Group-IQ as predictors of Moon Landing Exercise. The results indicated that none of the predictors significantly predicted Moon Landing Exercise. For Woolley’s *c*Factor, the regression equation was not significant, *F*(1, 27) = 0.35, *p* = 0.561, with an *R*^2^ of 0.01 (*β* = 1.03, *t*(27) = 0.59, *p* = 0.561). Similarly, for *c*Fluid, the regression equation was not significant, *F*(1, 27) = 0.30, *p* = 0.591, with an *R*^2^ of 0.01 (*β* = 0.95, *t*(27) = 0.54, *p* = 0.591). For *c*Crystal, the regression equation was not significant, *F*(1, 27) = 0.04, *p* = 0.835, with an *R*^2^ of 0.00 (*β* = 0.37, *t*(27) = 0.21, *p* = 0.835). Lastly, for Group-IQ, the regression equation was not significant, *F*(1, 27) = 0.35, *p* = 0.562, with an *R*^2^ of 0.01 (*β* = 1.03, *t*(27) = 0.59, *p* = 0.562).

We take this as evidence that collective intelligence, however it is operationalized, is unlikely to be a significant or substantial predictor of group performance on a complex criterion task. Various operationalizations of individual intelligence showed slightly higher correlations with the criterion task compared to collective intelligence (*r* ~ .21 vs. .08), but no values were statistically significant.

### Research question 4. Predicting group assignment grades

**Fig 5:**
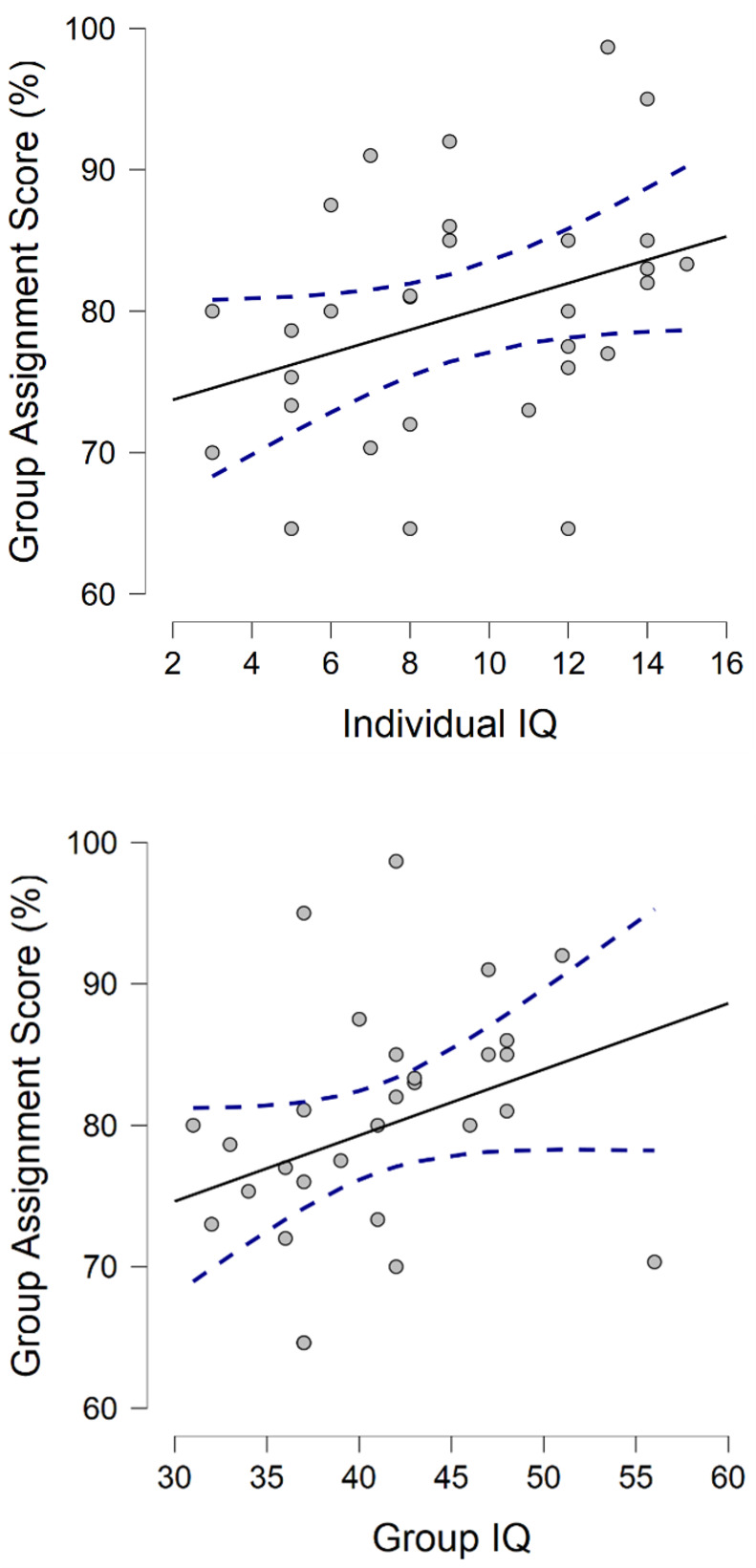
a and b. Individual-IQ vs. group-IQ and group assignment scores. ***a*.** Correlations between Individual IQ scores and Group Assignment Scores (%), ***b*.** Correlations between Group-IQ scores and Group Assignment Scores (%). ***Note*.** Results display Spearman’s rho, one-tailed. All correlations are considered only for the subset of participants objectively verified Group Assignment Scores (%) from their most recent university studies (n = 30).

The fourth research question (RQ4) explored whether collective intelligence had any predictive validity for group assignment grades received by students enrolled in higher education at the university-level. There was a moderate and statistically significant correlation between the participants’ individual IQ scores and their group assignment results, *r*_*s*_ = .40 [95% CI: .11, 1.0], *p =* .014 (Fig 5A). Similarly, we found a moderate and statistically significant correlation between the group-IQ composite scores and their group assignment results, *r*_*s*_ = .47 [95% CI: .19, 1.0], *p =* .005 (Fig 5B).

Following the results from research question 4 (RQ4), it appears that collective intelligence, as measured by group-IQ composite scores, shows moderate and statistically significant predictive validity for group assignment grades in higher education settings. This suggests that while collective intelligence may not significantly predict performance on immediate, complex tasks like the Moon Landing Exercise (as seen in RQ3), it does have relevance in academic contexts where collaborative efforts and sustained cognitive engagement over time are required. Notably, individual intelligence scores demonstrated nearly equally valid predictive power for group assignment results, indicating that both individual and collective cognitive abilities play significant roles in academic group performance. Therefore, the predictive power of collective intelligence might be more context-dependent, demonstrating utility in structured, educational group activities rather than in dynamic and immediate problem-solving scenarios. This distinction underscores the importance of considering the specific nature of the tasks when evaluating the effectiveness and applicability of collective intelligence measures, while also recognizing the substantial contribution of individual intelligence.

## Discussion

We set out to explore the role of collective intelligence among student groups. We did this by first exploring the factor structure of collective intelligence as it emerges from a battery of group problem-solving tasks, testing a subset of plausible causes and correlates of collective intelligence, and then seeing if our model of collective intelligence could predict group performance on a subsequent criterion task and on future group assignments in an Australian university setting. We also sought to add to the growing body of empirical research that clarifies whether collective is distinguishable from the intelligence of the groups’ individual membership. We organized our inquiry around answering four key questions, which we shall discuss in turn.

*Research Question 1*. Our competing model factor analysis favored a two- over a single-factor model. To our surprise, this two-factor model, while common among individual intelligence theorists, has not been considered in any previous study on collective intelligence. We referred to this as the *c*Fluid-*c*Crystal model (collective-fluid and collective-crystal) in light of two qualitatively distinct factors reflecting a dichotomy known to intelligence theorists for several decades [42, 49]. Cattell originally referred to this dichotomous model in a presentation to the American Psychological Association in 1941, and along with his doctoral student and future colleague, John Horn, developed what would become known as the Gf-Gc (fluid and crystallized general ability) model of human intelligence [42, 45, 46, 50]. In parallel with this theory, the first three sets of items in the present study loaded more on tasks involving rapid adaptation, novel problem-solving, and relied less on prior knowledge (*c*Fluid items), while the second two sets of items loaded more on tasks that harnessed the group’s accumulative knowledge and skills (*c*Crystal items). However, we note that *c*Fluid-*c*Crystal model may itself be a direct reflection of the underlying factor structure of the individuals within the group, rather than the group itself, and further research is needed to disambiguate these factors. One of the advantages of the present study that has been less obvious in prior research is the methodological decision to sample items from individual intelligence tests that have been psychometrically validated around the crystal-fluid dichotomy. These included fluid-reasoning items from Raven’s Progressive Matrices and its crystalized intelligence items from the Mill-Hill Vocabulary Scale [51] and Ekstrom’s Cognitive Ability Kit (advanced vocabulary section) [26].

While our *c*Crystal-*c*Fluid model remains a novel observation, our failure to support a single-factor model is not. Other studies have failed to support the single-factor model of collective intelligence proposed by Woolley et al. [9] and have instead observed findings consistent with a task-specific factor structure for group performance [14, 52–54]. An alternative view proposed by Bates and Gupta [16] *does* support a single-factor model governing group performance, but instead of the *c*-factor proposed by Woolley et al. [9], they suggest that the *g*-factor of the groups’ individual members is chiefly responsible for the common variance observed among group tasks. While our findings did not clearly favor the *c* or *g*-factor models, we did find some evidence to suggest that variation of intelligence at the group-level can be at least partly attributed to and thus nested within intelligence at the individual-level.

It is difficult to disambiguate the causal influences on collective intelligence in the present body of research because most of these studies have adopted methods used by Woolley et al. [9] where tasks were sampled from an organizational paradigm and not an IQ-testing paradigm [36]. It is therefore possible that such tests may only be of marginal relevance to the notion of intelligence as it relates to psychometric *g*, despite claims made by Woolley et al. [9] that *g* is the theoretical analogue of the *c*-factor. Similarly, the classification scheme employed in the review by Graf-Drasch et al. [14] also drew upon McGrath’s task circumplex, eventually condensing this into a two-tiered framework (*ill*-structured vs. *well*-structured) that shared parallels with Laughlin’s [55] ‘judgemental’ vs. ‘intellective’ task paradigm. Again, it is notable that they did not draw upon established IQ test theory grounded in highly sophisticated factor-based models of intelligence [49, 56]. Instead, most researchers have favored qualitative classification schemes from the business and managerial sciences [36, 55, 57]. This may seem like a peculiar demarcation between task types because the original intent of Woolley et al.’s [5] study was to use the *same* methods used for measuring individual intelligence and apply them to measuring intelligence in groups:

“We have used the statistical approach they developed for individual intelligence to systematically measure the intelligence of groups. Even though social psychologists and others have studied for decades how well groups perform specific tasks, they have not attempted to measure group intelligence in the *same* way individual intelligence is measured” ([9], p. 686, italics added).

Excepting their factor analyses, which is not at all unique to models of human intelligence [58], Woolley et al. [9] seem to have abandoned in many important respects the methods used to measure individual intelligence without explicitly recognizing this, opting for a vastly different task sampling paradigm with no traditional ties to IQ testing. Moreover, it is not obvious that their existing method of task sampling was exclusively or even especially germane to group activities. This is evinced by the fact that in their original study, Woolley et al. [9] had individuals complete “group tasks” under individual control conditions. This raises the question: Why go to the trouble of adapting individual intelligence testing methods for the factor-analysis and not for the group task selection, particularly when the entire repertoire of tasks used across these studies could just as easily have been completed by both individuals and groups, making the omission of existing individual IQ tests all the more peculiar.

It could be argued that the departure from the traditional methods and measures used for individual intelligence tests is not a trivial exception and on its own could explain away entirely the discordance between intelligence as measured at the group- (i.e., collective intelligence) and individual-levels (i.e., individual intelligence). These inconsistencies were raised in a study comprising two meta-analyses by Rowe et al. [15] who argued that using different IQ tests across the individual and group conditions “makes it entirely unclear how much of the total variance in group IQ tests that the *g*-factor accounted for at the group level” (p. 17). If Woolley et al. [9] were to remain true to the methods used to test individual intelligence, Rowe et al. [15] argue, they would select tasks along with the administration protocols that have been “psychometrically validated around various domains of mental ability, such as visual processing, reading and writing, and fluid reasoning” (p. 17) and not McGrath’s group task circumplex.

Future studies may seek to explore this possibility more thoroughly by using parallel sets of psychometrically validated IQ tests for both individual and group-conditions. By holding test-items constant across a multilevel structural equation model and using psychometrically validated IQ tests across individual and group conditions, researchers can more easily disentangle the nested effects of the *g* from the *c*-factor in group performance settings. This suggestion is especially notable given that one of our strongest correlations among predictor and outcome variables was between the individual-level IQ tests and the Raven’s matrices completed at the group-level. The review by Credé and Howardson [54] make a similar case using simulations of correlation matrices from previous studies. They tested the idea of ensuring the IQ tests administered at the individual-level were the same as those administered at the group-level and, once the individual-IQ data was accounted for, found little need to hypothesize a *c*-factor because there is no common variance left unexplained.

Brainstorming uses for an object, unscrambling words, matrix reasoning, typing numbers, brainstorming words, typing text, sudoku, and memorizing pictures make up the core battery of tasks in the Platform for Online Group Studies (POGS) that underpin the entire research program generated by Woolley and colleagues, as outlined in the meta-analysis by Riedl et al. [13]. It is not clear that any of these tasks are either psychometrically validated or strictly “group” tasks and, in fact, each of them seems to have been sampled from tasks that were originally designed for completion by individuals and not for the purpose of testing intelligence, with only one exception (matrix reasoning). Until researchers in this field can plausibly demarcate group-IQ tasks from individual-IQ tests, the entire edifice of the collective intelligence testing paradigm remains vulnerable to criticisms that the *c*-factor, when individual intelligence is properly accounted for, is but a mere statistical artefact of individual ‘*g*’ writ large. This was indeed the claim made by Bates and Gupta [16].

This unresolved issue has particularly important implications for educational, organisational, and workplace practices that rely heavily on groups to accomplish key tasks. Woolley and colleagues claim that the *c*-factor can be used to “predict a sales team’s or a top management team’s long-term effectiveness” ([5], p. 688), “companies might also give CI [collective intelligence] tests to their internal teams and use the results as early indicators to intervene in various ways” … “If a team performed poorly… managers might change some of the people on the team or provide external coaching. . . teams that performed well might be given more important assignments” ([9], p. 5). Our results challenge the claim that measures of collective intelligence should usurp existing measures that predict group performance (e.g., individual intelligence) because the *c*-factor needs to first be more thoroughly interrogated for its existence beyond psychometric *g* and in relation to alternative factor structures–points seldom considered in previous research. Until such work is undertaken by a range of independent researchers and to the highest standard, it remains premature to recommend such profound implications for educational or workplace practices.

*Research Question 2*. With respect to the correlates of collective intelligence, we did not find any significant relationship between the hypothesized causal variables proposed by Woolley et al. [9] (conversational turn-taking, social perceptiveness via Reading the Mind in the Eyes, and the percentage of women in the group). Importantly, our examination of the causes and correlates were considered in relation to three operationalizations of collective intelligence (raw group-IQ scores, *c*Fluid, and *c*Crystal factors). A secondary analysis found conscientiousness was moderately related to collective intelligence, especially when operationalized as the group-IQ composite scores and the *c*Crystal factor loadings. This corroborates prior research that indicates groups in a variety of settings (e.g., work, school) tend to perform better when they have members who possess higher levels of conscientiousness [59, 60]. Conscientiousness, in this instance, is a dimension of the five-factor model of personality that inclines one to behave in an organized, hardworking, and responsible manner. When considered in isolation of other influences on group performance, meta-analyses show that a groups’ average individual levels of conscientiousness (*r* = .14) or individual intelligence (*r* = .31) may be just as important as any emergent features of the group itself [61]. Other reviews find that, when coupled with IQ test outcomes, ratings of employee conscientiousness improve incremental predictive validity of job performance from *r =* .51 to .60 [62].

It remains unclear where the richest vein of process-related data lies in the evolution of research on teams [63], making the growing number of studies on collective intelligence especially intriguing because they provide important clues as to where to shine the proverbial spotlight. The present study was able to account for a small selection of communication-related processes (e.g., conversational turn-taking, word-count), but none shared a significant relationship with group performance. Had we administered the tasks using the POGS system developed by Riedl et al. [13] rather than using a paper-based testing system, a more nuanced account of the group processes could have been provided and future research would do well to pre-empt and capitalize on these opportunities.

*Research Questions 3 and 4*. The third and fourth research questions compared the predictive validity of individual and collective intelligence; the first in relation to a complex criterion task held immediately after the group-IQ test and the second in relation to group assignment grades from the participants’ university studies. To ensure the thoroughness of our investigation, we operationalized and analyzed collective intelligence across three levels (group-IQ scores, *c*Fluid and *c*Crystal loadings). Neither of these levels of analysis demonstrated any significant relationship with the complex criterion task, the group Moon Landing Exercise, and led us answer RQ3 in the negative and retain the null hypothesis. This was surprising given that the original and subsequent studies by Woolley et al. found the *c-*factor consistently shared a moderate to strong correlation with similar tasks [9, 12].

Further complicating our interpretation were the results from our correlational analysis between our two measures of intelligence (e.g., individual IQ and group-IQ scores) and participants’ group assignment results for a university-level study program. This part of the study was included to address issues surrounding the ecological validity of the criterion task (i.e., the Moon Landing Exercise), which may not have otherwise included the same abilities that would be most relevant to real-world group performance. Both the individual and group-IQ scores performed equally well and showed moderate to strong correlations with individual participants’ group assignment results. This suggests that both measures may have some merit in predicting team learning and group performance in real-world educational settings. While several major reviews have firmly established the connection between an individual’s intelligence and their ability to influence group performances [61, 64, 65], the same cannot be said of the notion of collective intelligence where the lineage of studies supporting its role in group performance are largely restricted to those published by Woolley and colleagues [15].

## Conclusions

We set out to examine the validity of a general collective intelligence factor, assess its underlying factor structure, and evaluate its predictive power on group problem-solving skills and academic outcomes. In working toward these ends, we were able to generate several unique theoretical and practical insights. Our results challenged the single-factor model (i.e., the *c*-factor) of collective intelligence by suggesting it may not be the only–or even the optimal way–to model the collective cognitive abilities of groups. We observed performance patterns across group tasks that paralleled those using psychometric theory in the study of individual intelligence, namely, Cattell’s crystal-fluid model of intelligence [46]. To the best of our knowledge the model that we propose, the *c*Fluid-*c*Crystal model, is the first in the group problem-solving and performance literature to suggest that such trends may scale from individual to group settings. The many broad and narrow factors that have been shown to influence psychometric *g* may provide a good starting point for researchers interested in mapping out a more nuanced perspective on collective intelligence and expanding on the line of reasoning commenced in this study.

In addition, researchers need to examine whether various sub-domains of collective intelligence either emerge from or are separate to the intelligence of the groups’ individual membership. It remains possible that the crystal-fluid pattern that we observed was simply a reflection of the characteristics of intelligence observed in the groups’ individual members. Until such matters can be resolved, it may be worth tempering expectations about the potential for group-IQ tests to replace individual-IQ tests as measures of group performance in various educational and occupational settings. This was supported by our observation that measures of individual ability (e.g., IQ tests) predict group assignment scores equally well compared to measures of collective intelligence (e.g., group-IQ tests). In such contexts, where incremental validity is awash, it could be argued that a more sensible approach is to utilize individual-level measures of ability because they are situated within a unified cognitive agent, an individual person, whose mental properties are relatively stable across a lifetime [66]. The same cannot necessarily be said for measures of collective intelligence where the unit of analysis (e.g., small groups) is inherently unstable due to issues such as member turnover, multiteam membership, social loafing, low engagement, absenteeism, ostracization, up or downsizing, conflicts, and political factionalism.

Finally, our study raises questions about the main variables thought to be involved in fostering collective intelligence in groups. Unlike several studies by Woolley and colleagues [9, 10, 67], we found little to no evidence affirming the importance of the group’s conversational turn-taking, social perceptiveness as measured by the Reading the Mind in the Eyes test, or the proportion of women in the group. Our secondary analysis hinted at the possibility that alternative composition variables, such as conscientiousness and individual intelligence, may play an important role in cultivating collectively intelligent small groups; but these findings were incidental to the main analyses and have already been established in the literature as fundamental drivers of group performance [60, 61].

## Limitations and future directions

The new paradigm of collective intelligence advocated for by Woolley et al. [9] is a fascinating and implication-laden construct for group learning, problem-solving, and performance. It nevertheless remains an underdeveloped field that is beset with methodological shortcomings and a lack of data over longitudinal periods–which can significantly alter group performance in real-world conditions [31, 68]. Future research would benefit from considering such interactions in the context of studies spanning months or even years.

Our study was somewhat limited by the face-to-face and paper-based testing context we employed, which made it difficult to capture some potentially important process-related data based on effort and time-on-task, conflict, voice tone, body-language, facial expressions, planning and decision-making, and task-level engagement. In future studies, it may prove more fruitful for researchers to build their collective intelligence testing program in digitalized environments, where it is easier to collect a rich variety of ‘thick’ data, perhaps by adapting the Platform for Online Group Study (POGS) proposed by Riedl et al. [13].

Another potential shortcoming is that the subset of participants who shared their group assignment results with us may have been more likely to have favorable academic results compared to those who received but chose to withhold their group assignment scores. This ‘social desirability’ bias is a well-known confound in the social sciences [69]. Future studies could secure the ethical clearance necessary to attain academic results without having to trouble the individual participants to manually retrieve and share their data with researchers (e.g., by accessing a university results database).

Finally, studies need more innovative and less blunt instruments to account for the within-group and between-group variability of individual intelligence and other individual-level measures. This has important implications for research that seeks to disentangle the influence of individual and collective intelligence in group tasks, but also applies to other “group-level” variables that are derived from averaging individual-level data. A promising example involves weighting the potential contributions from individuals to group tasks by benchmarking performances on a series of control tasks or calibrating estimated contributions based on an individual’s prior academic achievement or cognitive ability [70].

## Supporting information

S1 File Data group phase–Main analysis. (CSV)

S2 File Data group phase–Secondary analysis. (CSV)

S3 File Participant demographics. (PDF)

S4 File Additional analyses. (PDF)
