# Supplementary material for: High-performing teams: Is collective intelligence the answer?
Source: PLoS One. 2024 Aug 12;19(8):e0307945. doi: 10.1371/journal.pone.0307945 (PMC11318883; doi:10.1371/journal.pone.0307945)
Supplement: S3 File — (PDF) [file pone.0307945.s003.pdf]

Frequency Tables

Frequencies for Age

Age	Frequency	Percent	Valid Percent	Cumulative Percent
18	2	2.353	2.353	2.353
19	3	3.529	3.529	5.882
20	6	7.059	7.059	12.941
21	16	18.824	18.824	31.765
22	13	15.294	15.294	47.059
23	10	11.765	11.765	58.824
24	7	8.235	8.235	67.059
25	3	3.529	3.529	70.588
26	4	4.706	4.706	75.294
27	3	3.529	3.529	78.824
28	5	5.882	5.882	84.706
31	1	1.176	1.176	85.882
32	1	1.176	1.176	87.059
33	2	2.353	2.353	89.412
34	2	2.353	2.353	91.765
35	2	2.353	2.353	94.118
37	1	1.176	1.176	95.294
42	1	1.176	1.176	96.471
50	1	1.176	1.176	97.647
54	1	1.176	1.176	98.824
60	1	1.176	1.176	100.000
Missing	0	0.000		
Total	85	100.000		

Frequencies for Main_Language

Main_Language	Frequency	Percent	Valid Percent	Cumulative Percent
English	35	41.176	41.176	41.176
Non-English	50	58.824	58.824	100.000
Missing	0	0.000		
Total	85	100.000		

Frequencies for Edu_Attain

Edu_Attain	Frequency	Percent	Valid Percent	Cumulative Percent
Certificate I/II/III/IV	2	2.353	2.353	2.353
Diploma	5	5.882	5.882	8.235
Four Year University Degree	25	29.412	29.412	37.647
High School Certificate	18	21.176	21.176	58.824
Masters Degree	16	18.824	18.824	77.647
Secondary School	3	3.529	3.529	81.176
Three Year University Degree	16	18.824	18.824	100.000
Missing	0	0.000		
Total	85	100.000		

Frequencies for CoB

CoB	Frequency	Percent	Valid Percent	Cumulative Percent
Australian Born	14	16.471	16.471	16.471
Overseas Born	71	83.529	83.529	100.000
Missing	0	0.000		
Total	85	100.000		

Frequencies for Occupation

Occupation	Frequency	Percent	Valid Percent	Cumulative Percent
Currently Unemployed	1	1.176	1.176	1.176
Full-time Student	82	96.471	96.471	97.647
Other	1	1.176	1.176	98.824
Part-time Employed (or casual)	1	1.176	1.176	100.000
Missing	0	0.000		
Total	85	100.000		

Frequencies for Ethnicity

Ethnicity	Frequency	Percent	Valid Percent	Cumulative Percent
North African and Middle Eastern	1	1.176	1.176	1.176
North-East Asian	7	8.235	8.235	9.412
North-West European	14	16.471	16.471	25.882
Oceanian	2	2.353	2.353	28.235
Other	1	1.176	1.176	29.412
People of Americas	1	1.176	1.176	30.588
South-East Asian	34	40.000	40.000	70.588
Southern and Central Asian	22	25.882	25.882	96.471
Southern and Eastern European	3	3.529	3.529	100.000
Missing	0	0.000		
Total	85	100.000		

Frequencies for Sex

Sex	Frequency	Percent	Valid Percent	Cumulative Percent
Female	61	71.765	71.765	71.765
Male	24	28.235	28.235	100.000
Missing	0	0.000		
Total	85	100.000		
