# Supplementary material for: High-performing teams: Is collective intelligence the answer?
Source: PLoS One. 2024 Aug 12;19(8):e0307945. doi: 10.1371/journal.pone.0307945 (PMC11318883; doi:10.1371/journal.pone.0307945)
Supplement: S4 File — (PDF) [file pone.0307945.s004.pdf]

Analyses not included in the manuscript.

Parallel Analysis:

```
> paran(CIGroup_EFA, cfa=TRUE)

Using eigendecomposition of correlation matrix.
Computing: 10%  20%  30%  40%  50%  60%  70%  80%  90%  100%


Results of Horn's Parallel Analysis for factor retention
150 iterations, using the mean estimate

--------------------------------------------------
Factor      Adjusted    Unadjusted    Estimated
            Eigenvalue  Eigenvalue    Bias
--------------------------------------------------
No components passed.
```

1	0.674512	1.392721	0.718208
2	0.093156	0.423555	0.330399
3	0.221753	0.287991	0.066237

Adjusted eigenvalues > 0 indicate dimensions to retain.

(3 factors retained)

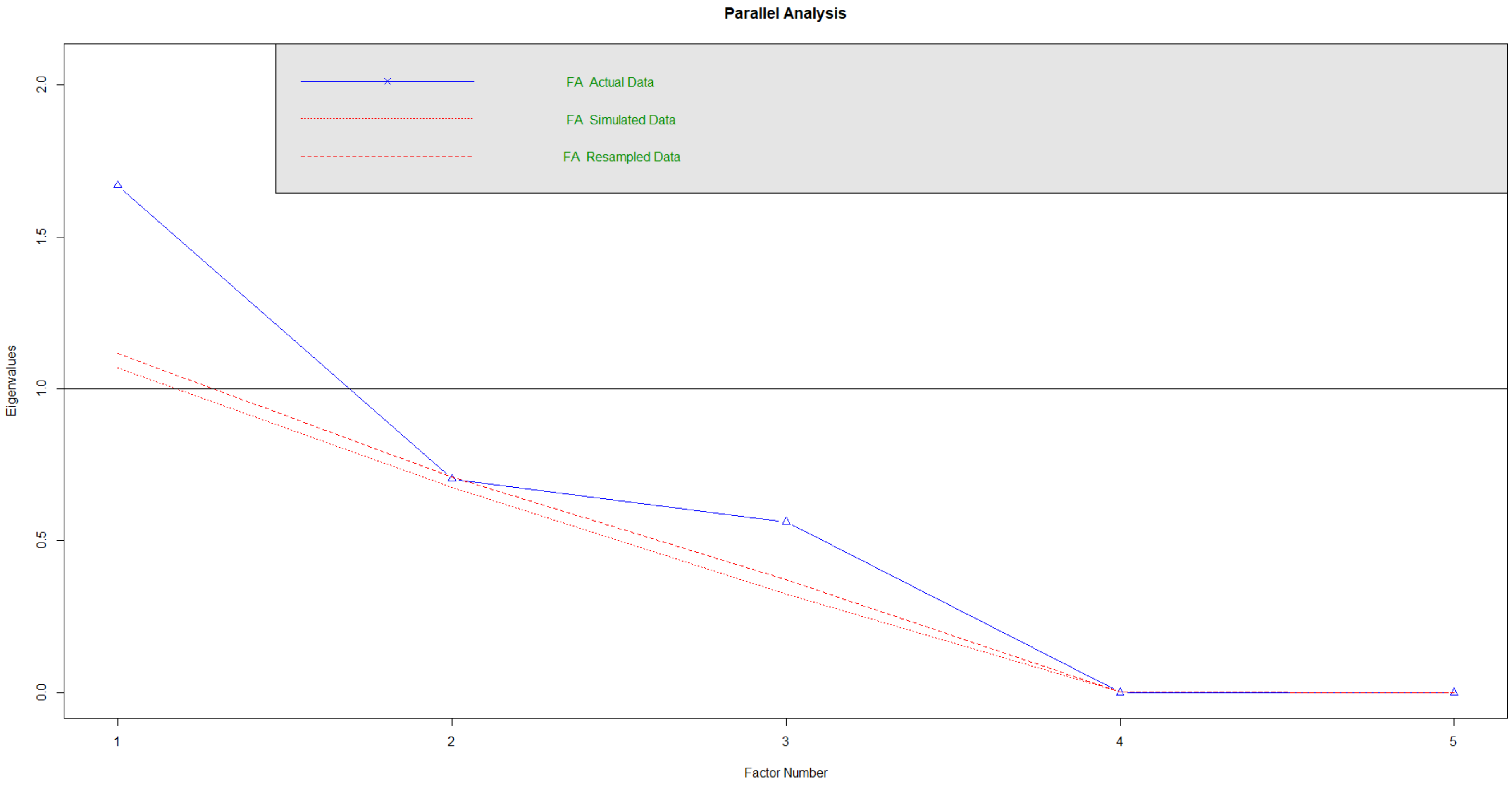

Parallel Analysis
FA Actual Data
FA Simulated Data
FA Resampled Data
Eigenvalues
0.0
0.5
1.0
1.5
2.0
1
2
3
4
5
Factor Number

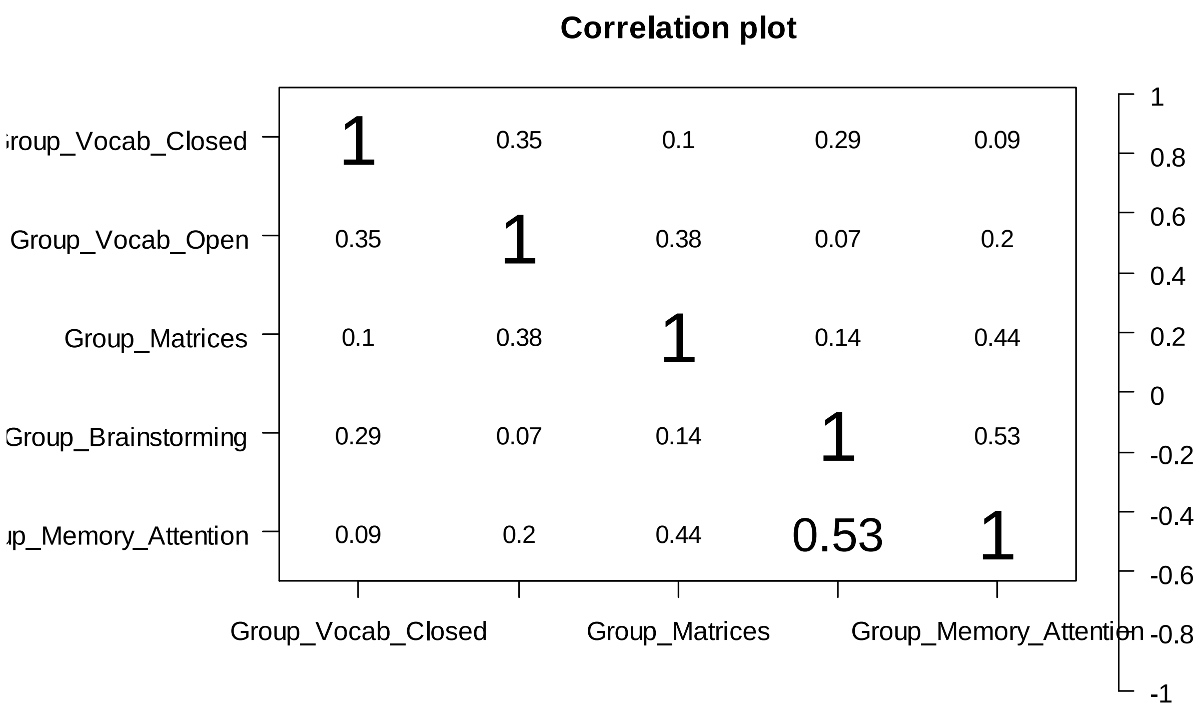
Correlation plot
roup_Vocab_Closed
Group_Vocab_Open
Group_Matrices
Group_Brainstorming
p_Memory_Attention
1 0.35 0.1 0.29 0.09
0.35 1 0.38 0.07 0.2
0.1 0.38 1 0.14 0.44
0.29 0.07 0.14 1 0.53
0.09 0.2 0.44 0.53 1
Group_Vocab_Closed
Group_Matrices
Group_Memory_Attention
1
0.8
0.6
0.4
0.2
0
-0.2
-0.4
-0.6
-0.8
-1

Power Calculations:

#calculate sample size with two-tailed tolerance for correlation coefficients of r = 0.52, p <= .05, power >= 80%

> pwr.r.test(n = , r = 0.52, sig.level = .05, power = .80)

approximate correlation power calculation (arctangh transformation)

n = 25.87738

r = 0.52

sig.level = 0.05

power = 0.8

alternative = two.sided

> #calculate sample size with one-tailed tolerance for correlation coefficients of r = 0.52, p <= .05, power >= 80%

> pwr.r.test(n = , r = 0.52, sig.level = .05, power = .80, alternative="greater")

approximate correlation power calculation (arctangh transformation)

n = 20.74515

r = 0.52

```
     sig.level = 0.05
         power = 0.8
   alternative = greater

>
> # Plot sample size curves for detecting correlations of
> # various sizes.
>
> # range of correlations
> r <- seq(.1,.5,.01)
> nr <- length(r)
>
> # power values
> p <- seq(.4,.9,.1)
> np <- length(p)
>
> # obtain sample sizes
> samsize <- array(numeric(nr*np), dim=c(nr,np))
> for (i in 1:np){
+   for (j in 1:nr){
```

```
+    result <- pwr.r.test(n = NULL, r = r[j],
+                       sig.level = .05, power = p[i],
+                       alternative = "one.sided")
+    samsize[j,i] <- ceiling(result$n)
+  }
+ }
>
> # set up graph
> xrange <- range(r)
> yrange <- round(range(samsize))
> colors <- rainbow(length(p))
> plot(xrange, yrange, type="n",
+     xlab="Correlation Coefficient (r)",
+     ylab="Sample Size (n)" )
>
> # add power curves
> for (i in 1:np){
+  lines(r, samsize[,i], type="l", lwd=2, col=colors[i])
+ }
>
```

```
> # add annotation (grid lines, title, legend)
> abline(v=0, h=seq(0,yrange[2],50), lty=2, col="grey89")
> abline(h=0, v=seq(xrange[1],xrange[2],.02), lty=2,
+       col="grey89")
> title("Sample Size Estimation for Correlation Studies\n
+   Sig=0.05 (One-tailed)")
> legend("topright", title="Power", as.character(p),
+       fill=colors)
```

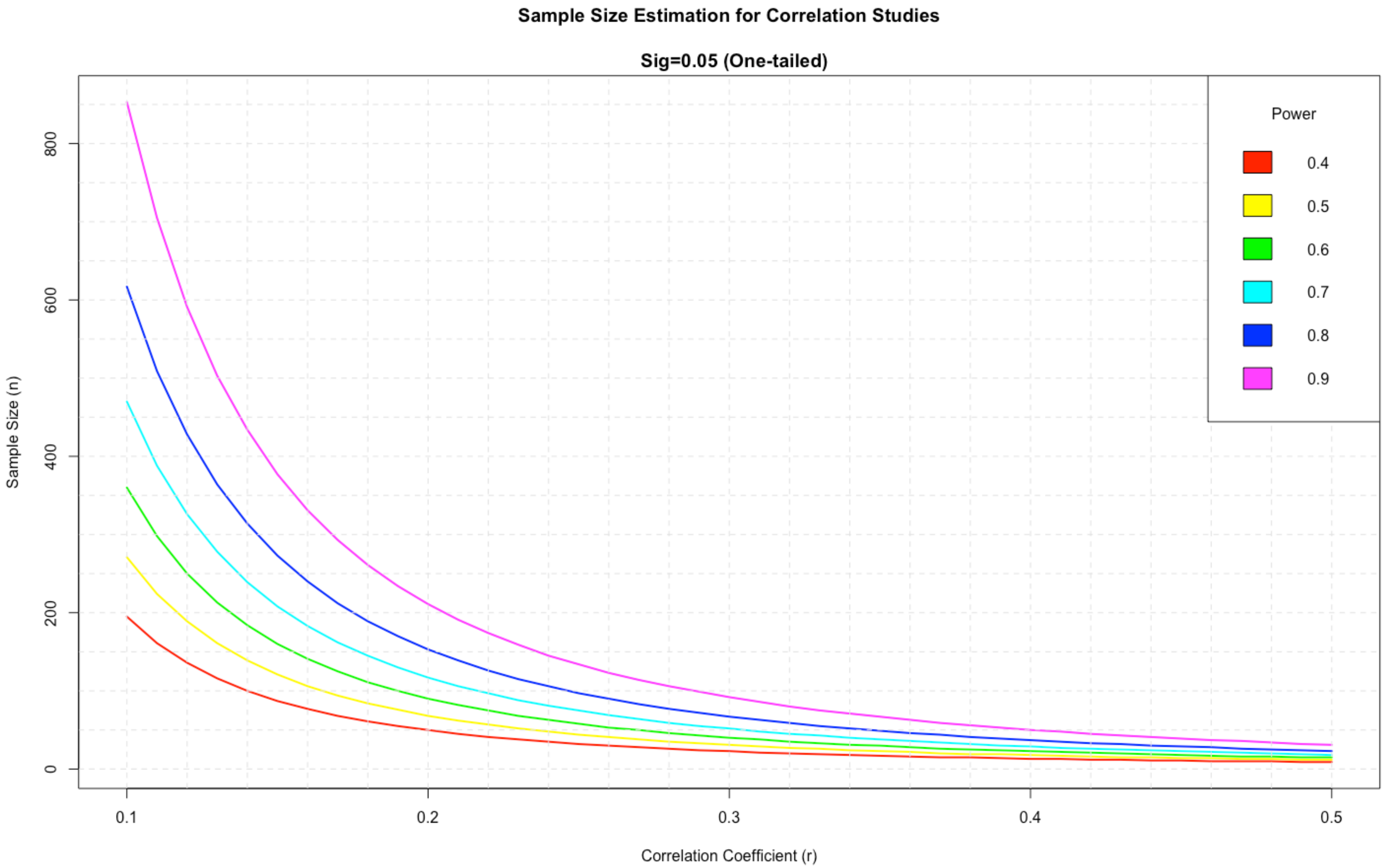
Sample Size Estimation for Correlation Studies
Sig=0.05 (One-tailed)
Sample Size (n)
0
200
400
600
800
0.1
0.2
0.3
0.4
0.5
Correlation Coefficient (r)
Power
0.4
0.5
0.6
0.7
0.8
0.9
